# NOX2 inhibition reduces oxidative stress and prolongs survival in murine KRAS*-*induced myeloproliferative disease

**DOI:** 10.1038/s41388-018-0528-1

**Published:** 2018-10-15

**Authors:** Ebru Aydin, Alexander Hallner, Hanna Grauers Wiktorin, Anna Staffas, Kristoffer Hellstrand, Anna Martner

**Affiliations:** 1 TIMM Laboratory, Sahlgrenska Cancer Center, University of Gothenburg, 413 90 Gothenburg, Sweden; 2 Department of Clinical Chemistry and Transfusion Medicine, Sahlgrenska Academy, University of Gothenburg, 413 45 Gothenburg, Sweden

**Keywords:** Myeloproliferative disease, Cancer genetics

## Abstract

Mutations leading to constitutive RAS activation contribute in myeloid leukemogenesis. *RAS* mutations in myeloid cells are accompanied by excessive formation of reactive oxygen species (ROS), but the source of ROS and their role for the initiation and progression of leukemia have not been clearly defined. To determine the role of NOX2-derived ROS in RAS-driven leukemia, double transgenic LSL-*Kras*^G12D^ × Mx1-*Cre* mice expressing oncogenic KRAS in hematopoietic cells (M-*Kras*^G12D^) were treated with *N*^α^-methyl-histamine (*N*MH) that targeted the production of NOX2-derived ROS in leukemic cells by agonist activity at histamine H_2_ receptors. M-*Kras*^G12D^ mice developed myeloid leukemia comprising mature CD11b^+^Gr1^+^ myeloid cells that produced NOX2-derived ROS. Treatment of M-*Kras*^G12D^ mice with *N*MH delayed the development of myeloproliferative disease and prolonged survival. In addition, *N*MH-treated M-*Kras*^G12D^ mice showed reduction of intracellular ROS along with reduced DNA oxidation and reduced occurence of double-stranded DNA breaks in myeloid cells. The in vivo expansion of leukemia was markedly reduced in triple transgenic mice where KRAS was expressed in hematopoietic cells of animals with genetic NOX2 deficiency (*Nox2*^−/−^ × LSL-*Kras*^G12D^ × Mx1-*Cre*). Treatment with *N*MH did not alter in vivo expansion of leukemia in these NOX2-deficient transgenic mice. We propose that NOX2-derived ROS may contribute to the progression of KRAS-induced leukemia and that strategies to target NOX2 merit further evaluation in *RAS*-mutated hematopoietic cancer.

## Introduction

RAS proteins are GTPases that transmit growth factor receptor signals to several downstream effector pathways, including the PI3 kinase (PI3K)-AKT and the RAF-MEK-ERK pathways, to trigger the proliferation, survival, growth, and differentiation of cells. In its active form, RAS is loaded with GTP, but the intrinsic GTPase activity of wild-type (WT) RAS leads to self-inactivation once the growth factor signal ceases. *RAS* mutations resulting in loss of GTPase function entail constitutive, non-growth factor-dependent RAS signaling that contributes to malignant transformation. RAS mutations leading to constitutive RAS activation are observed in ~ 30% of cancer patients [1] and in 15% of patients with hematopoietic malignancies [2].

*KRAS* is one of three homologues of the *RAS* family of oncogenes in addition to *NRAS* and *HRAS*. Although these isoforms encode similar proteins, different variants of mutated *RAS* dominate, for unknown reasons, in different forms of cancer [3, 4]. *HRAS* mutations are thus rarely found in myeloid malignancies, whereas mutations leading to oncogenic *KRAS* or *NRAS* activation occur in ~ 40% of cases with chronic myelomonocytic leukemia [5] and in 20% of cases of monocytic (FAB classes M4 and M5) forms of acute myeloid leukemia (AML) [5–7].

Compounds that covalently attach KRAS G12C [8], antagonists of RAS-membrane association and downstream effector signaling [9, 10] and strategies to target downstream signaling of *KRAS* by inhibition of PI3K/Akt or Raf/MEK/ERK have shown promise in preclinical models of RAS-induced cancer [11–14]. For the present study, we asked if the production of reactive oxygen species (ROS), a downstream event that appears to be enhanced by RAS signaling, may contribute in RAS-induced leukemogenesis. Although earlier studies show that *RAS* mutations trigger enhanced ROS levels [15–21], the contribution by ROS generated during mitochondrial respiration or by the enzymatic formation of ROS via the NADPH oxidase (NOX) isoforms NOX1, NOX2, or NOX4 remains controversial [15, 17, 22–24].

To address the role of NOX2, which is the dominant source of enzymatically derived ROS in normal and leukemic myeloid cells [25–28], in KRAS-driven leukemia we utilized double transgenic LSL-*Kras*^G12D^ × Mx1-*Cre* mice where hematopoiesis was biased toward the NOX2^+^ granulocyte/monocyte linage. We also created triple transgenic *Nox2*^−/−^ × LSL-*Kras*^G12D^ × Mx1-*Cre* mice that were devoid of NOX2-dependent ROS formation. The double and triple transgenic mice were treated with *N*^α^-methyl-histamine (*N*MH) that inhibits NOX2 activity via agonist activity at histamine type 2 receptors (H_2_Rs). *N*MH is a major histamine metabolite with reduced activity at histamine type 1 receptors (H_1_Rs) and increased affinity at type 2 and type 3 receptors [29]. Our results imply a role for NOX2 in KRAS-induced myeloproliferation and suggest that pharmacological inhibition of NOX2 reduces oxidative stress to maintain genomic stability in *Kras*-mutated cells.

## Results

### LSL-*Kras*^*G12D*^ *×* Mx1-*Cre* mice develop myeloproliferative disease comprising mature CD11b^+^Gr1^+^ myeloid cells

**Fig. 1:**
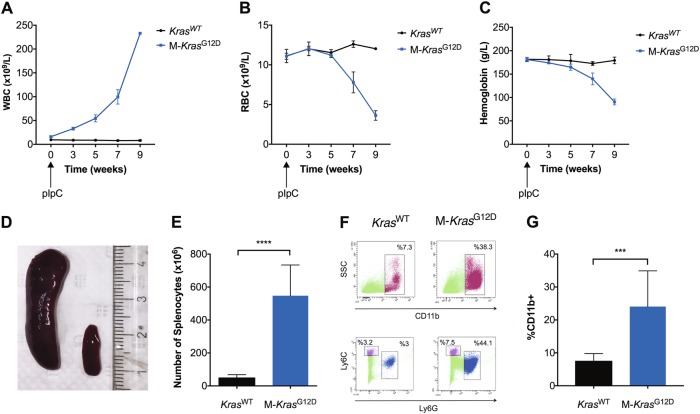
Characterization of *LSL-Kras*^G12D^ *×* Mx1*-Cre*-induced leukemia. **a**–**c** LSL-*Kras*^G12D^ *×* Mx1-*Cre* (M-*Kras*^G12D^; blue lines) mice and wild-type (*Kras*^WT^; black lines) mice were injected with pIpC at the time point w_0_. Peripheral blood was collected at 3, 5, 7, and 9 weeks (w3–9) after pIpC injection. The peripheral blood of M-*Kras*^G12D^ mice showed a progressive increase in white blood cell (WBC) counts **a**, along with a reduction of red blood cell (RBC) counts **b** and hemoglobin levels (**c**; *n* = 4–9 for *Kras*^WT^, *n* = 15–26 for M-*Kras*^G12D^). **d** Representative spleens from an endpoint M-*Kras*^G12D^ mouse (left) and a *Kras*^WT^ mouse (right). **e** Number of splenocytes isolated from endpoint M-*Kras*^G12D^ mice (blue, *n* = 17). The splenocyte counts from *Kras*^WT^ mice (black, *n* = 10) are shown for comparison (*t-*test). **f** FACS plots showing expansion of CD11b^+^ Ly6G^+^ and Ly6C^+^ myeloid cells in the spleen of a representative endpoint M-*Kras*^G12D^ mouse and a *Kras*^WT^ mouse. **g** Frequency of CD11b^+^-infiltrating cells among live cells in the spleens of endpoint M-*Kras*^G12D^ mice (blue, *n* = 10) and *Kras*^WT^ mice (black, *n* = 8) (*t* test). *p < 0.05, **p < 0.01, ***p < 0.001

LSL-*Kras*^G12D^ and Mx1-*Cre* mice were mated to generate double transgenic LSL-*Kras*^G12D^ *×* Mx1*-Cre* (M-*Kras*^G12D^) pups. *Kras*^G12D^ expression was induced in 3–6-week old M-*Kras*^G12D^ pups by polyinosinic–polycytidylic acid (pIpC) injections. In accordance with previous studies [30] the white blood cell (WBC) levels rose beginning ~ 3 weeks after pIpC injections followed by a significant drop in red blood cell (RBC) counts and in hemoglobin levels (Fig. 1a–c). At the study endpoint, i*.*e., ~ 3–10 weeks after pIpC injections, the spleens of the M-*Kras*^G12D^ mice were markedly enlarged compared with spleens of WT littermates (Fig. 1d). The splenomegaly translated into high counts of splenocytes in M-*Kras*^G12D^ mice (Fig. 1e) and spleens of M-*Kras*^G12D^ mice were infiltrated by CD11b^+^Gr1^+^ myeloid cells (Fig. 1f, g). The bone marrow of endpoint M-*Kras*^G12D^ mice contained a higher percentage of CD11b^+^Gr1^+^ cells compared with WT littermates (*p* = 0.03, *n* = 15 for each group. *t* test). Although all mice showed signs of myeloproliferative disease, ~ 40% of the M-*Kras*^G12D^ mice also developed T-cell leukemia, as determined by presence of CD4 and CD8 double-positive T cells along with enlarged thymuses (data not shown).

### *N*MH inhibits ROS formation from myeloid M-*Kras*^G12D^ cells

**Fig. 2:**
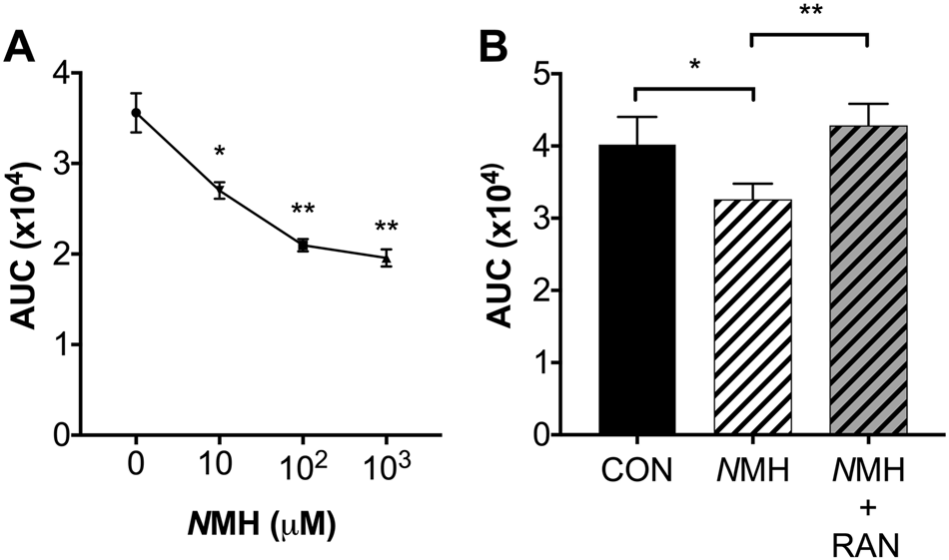
*N*MH inhibits ROS production by Gr1^+^ cells from M-*Kras*^G12D^ mice. **a** Two-hundred thousand Gr1^+^ cells from the spleen of M-*Kras*^G12D^ mice were stimulated with WKYMVm (5×10^−7^M) in the presence or absence of *N*MH at indicated concentrations. Superoxide anion production was measured continuously during 12 min by chemiluminescence and is displayed as area under the curve (AUC) (*n* = 3 for each concentration). **b** Superoxide production from Gr1^+^ cells from the spleens of M-*Kras*^G12D^ mice in response to stimulation with WKYMVm (5×10^−7^ M) in the presence or absence of *N*MH (100 µM) and the H_2_R antagonist ranitidine (RAN; 100 µM) (*n* = 4 for each group, *t* test). *p < 0.05, **p < 0.01, ***p < 0.001

CD11b^+^Gr1^+^ myeloid cells isolated from diseased M-*Kras*^G12D^ mice were stimulated with the NOX2-inducer WKYMVm [31] in the presence or absence of *N*MH for analysis of superoxide anion, monitored continuously by chemiluminescence. It was observed that CD11b^+^Gr1^+^ cells produced significant amounts of superoxide anion in response to WKYMVm, which was dose-dependently targeted by *N*MH (Fig. 2a). *N*MH exerts selectively agonist activity at H_2_ receptor (H_2_R) [29] and ranitidine, a H_2_R-specific antagonist [32], completely prevented the inhibitory effect of *N*MH on WKYMVm-induced superoxide anion formation (Fig. 2b).

### *N*MH delays the development of myeloproliferative disease and prolongs the survival of M-*Kras*^G12D^ mice

**Fig. 3:**
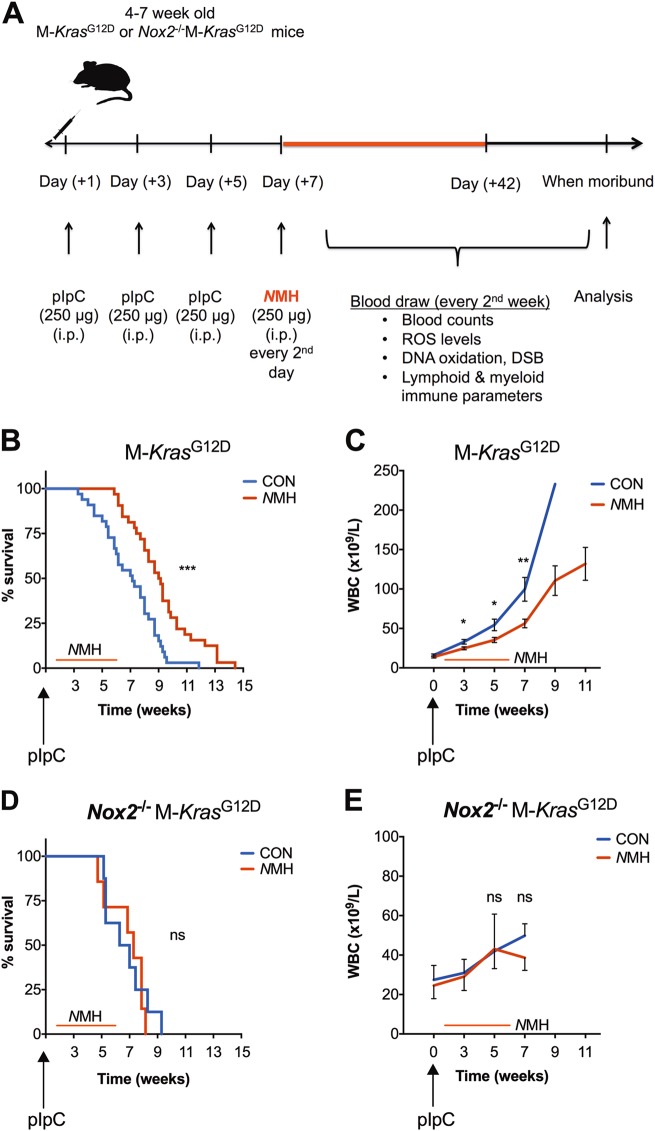
*N*MH delays the development of leukemia in NOX2-sufficent M-*Kras*^G12D^ mice. **a** shows the experimental design of the *in vivo* experiments. **b–c** *Kras* expression in hematopoietic cells (M-*Kras*^G12D^) was induced in double transgenic mice (LSL-*Kras*^G12D^ × Mx1-*Cre*) by pIpC injections. Mice were treated with *N*MH (red line) or NaCl (CON; blue line) i.p. thrice weekly for 5 weeks. **b** Survival of control (blue line) and *N*MH-treated (red line) mice (*n* = 28 for *N*HM, *n* = 29 for controls; logrank test). **c** White blood cell counts (WBC) in peripheral blood during disease progression in *N*MH-treated (red line) and control (blue line) mice (*n* = 26 for each group, *t* test). **d**, **e** *Kras* expression was induced in triple transgenic mice (*Nox2*^−/−^, LSL-K*ras*^G12D^, and Mx1-*Cre*) denoted *Nox2*^−/−^ M-*Kras*^G12D^ mice. Mice were treated with *N*MH (red line) or NaCl (CON; blue line) i.p. thrice weekly. **d** Survival of control (blue line) and *N*MH-treated (red line) triple transgenic mice (*n* = 8 for controls and *n* = 7 for the *N*MH-treated group; logrank test). **e** Counts of white blood cells in blood during progression of disease of control (blue line) and *N*MH-treated (red line) mice (*n* = 8 for controls and *n* = 7 for *N*MH-treated mice; *t* test). *p < 0.05, **p < 0.01, ***p < 0.001

One week after the first pIpC injection, M-*Kras*^G12D^ mice were randomized to receive intraperitoneal (i.p.) injections of *N*MH (250 μg/mouse) or vehicle (NaCl) thrice weekly for 5 weeks (Fig. 3a). Mice receiving *N*MH experienced a milder course of disease with better mobility, fur condition, and posture compared with control animals (data not shown). Treatment with *N*MH also improved the survival of M-*Kras*^G12D^ mice (Fig. 3b) and delayed the development of myeloid leukemia (Fig. 3c). While on treatment, 3 out of 28 M-*Kras*^G12D^ mice in the *N*MH-group died of leukemia versus 14/29 mice in the control group (*p* = 0.003, Fisher’s exact test).

### Role of NOX2 for the anti-leukemic efficacy of *N*MH in M-*Kras*^G12D^ mice

To further clarify the role of NOX2 for the observed effects of *N*MH on the development of *Kras*-induced leukemia and survival, we generated triple transgenic mice that expressed the Mx1-*Cre*-inducible LSL-*Kras*^G12D^ on a NOX2-deficient background (*Nox2*^−/−^). For these experiments, *Nox2*^−/−^ mice were mated with LSL-*Kras*^G12D^ and Mx1-*Cre* mice followed by at least three backcrosses. The knockout of the *Nox2* gene was confirmed by genotyping and by the absence of NOX2-dependent superoxide production (Supplementary figure 1A, B).

We observed significant myeloproliferation and anemia in blood of the triple transgenic *Nox2*^−/−^ M-*Kras*^G12D^ mice, suggesting that leukemia was successfully induced also in the absence of NOX2 (Supplementary figure 2). The increase in WBC counts was however markedly reduced in untreated *Nox2*^−/−^ M-*Kras*^G12D^ vs. *Nox2*^+/+^ M-*Kras*^G12D^ mice (*p* = 0.02 at 5 weeks, *p* = 0.003 at 7 weeks, *t* test; Fig. 3c, e). In vivo treatment with *N*MH did not delay or reduce the development of leukemia in the triple transgenic *Nox2*^−/−^ mice (Fig. 3d, e). Knockout of *Nox2* did not translate into a survival benefit in M-*Kras*^G12D^ mice. The triple transgenic mice showed increased susceptibility to infection in the form of abscesses in skin, liver or lungs. Visible infections were thus observed in ~ 45% of *Nox2*^−/−^ M-*Kras*^G12D^ mice during the course of the study, compared with 3% of double transgenic *Nox2*^+/+^ M-*Kras*^G12D^ mice and 3% of *Nox2*^−/−^*Kras*^WT^ mice. The incidence of visible infections was not significantly affected by *N*MH treatment (data not shown).

### NOX2 inhibition reduces intracellular ROS levels in myeloid cells

**Fig. 4:**
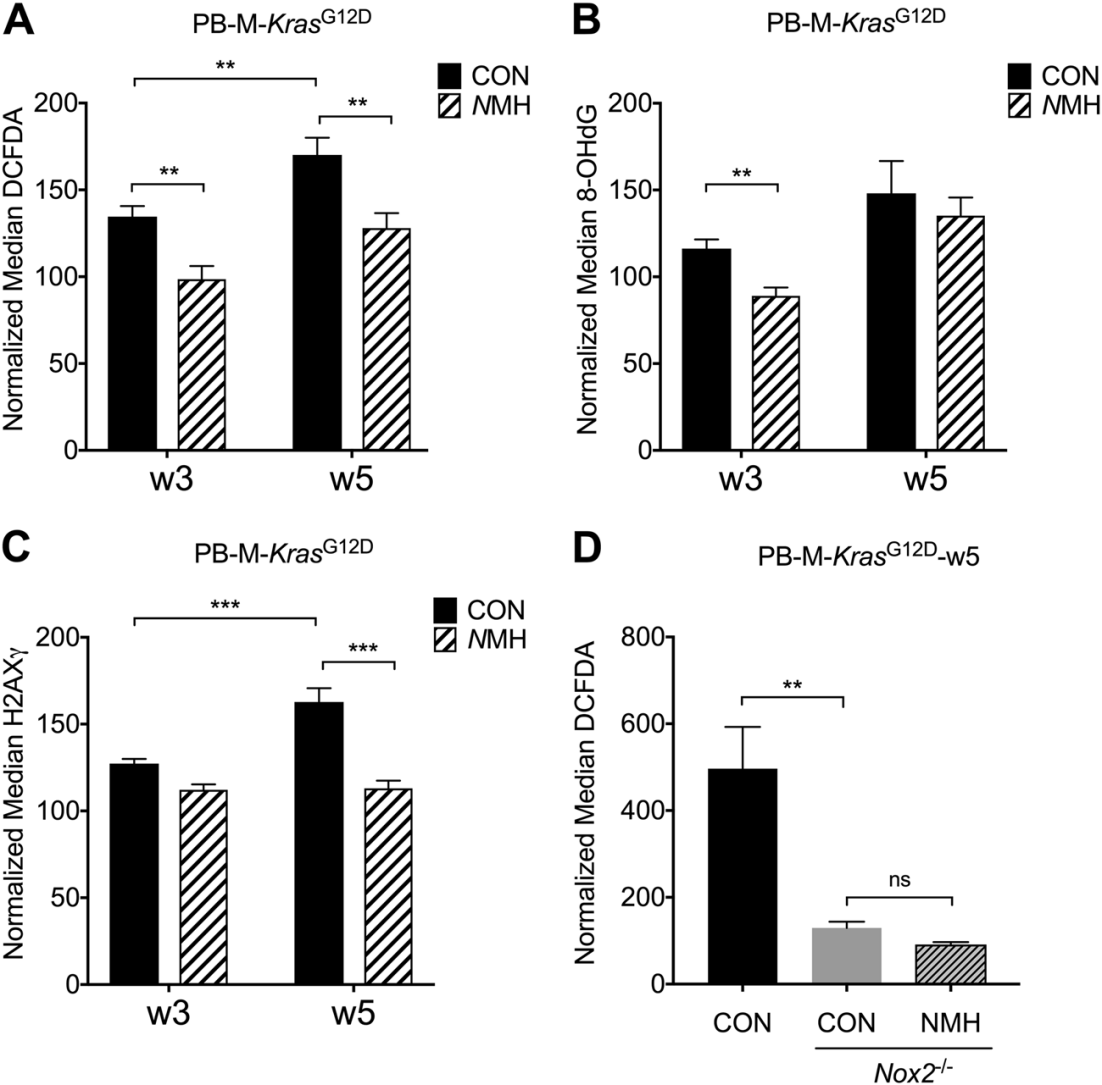
Treatment of mice with *N*MH maintains genomic integrity. Expression of mutated *Kras* in hematopoietic cells was induced in **a**–**c** double transgenic mice (LSL-*Kras*^G12D^ × Mx1-*Cre*; M-*Kras*^G12D^) and **d** triple transgenic mice (*Nox2*^−/−^, LSL-*Kras*^G12D^, and Mx1-*Cre*; *Nox2*^−/−^ M-*Kras*^G12D^) by pIpC injections. Mice were treated with *N*MH (250 μg/mouse; red) or NaCl (CON; blue) i.p. thrice weekly for 5 weeks. For M-*Kras*^G12D^ mice, peripheral blood samples collected after 3 and 5 weeks (w3, w5) were analyzed for **a** intracellular ROS levels in CD11b^+^ cells by use of DCFDA staining (*n* = 5–7), **b** oxidized DNA by measurement of 8-OHdG expression in myeloid cells (*n* = 7–8), and **c** double-stranded DNA breaks reflected by the expression of gamma-H2AX in myeloid cells (*n* = 7–8 for each week and each group). In **a**–**c**, the stainings for DCFDA, 8-OHdG, and gamma-H2AX were normalized against staining of myeloid cells in blood of *Kras*^WT^ mice. The increases in staining levels between w3–w5 were analyzed by Student's paired *t* test, whereas differences between control and NMH-treated samples were analyzed by Student's *t* test. **d** For *Nox2*^−/−^ M-*Kras*^G12D^ mice, peripheral blood samples were collected 5 weeks after pIpC and analyzed for intracellular ROS levels in CD11b^+^ cells by use of DCFDA (*n* = 5 for controls, *n* = 4 for *N*MH-treated mice). Peripheral blood collected 5 weeks after pIpC from M-*Kras*^G12D^ mice (*n* = 5) was analyzed in parallel, and the stainings were normalized against DCFDA staining of myeloid cells in blood of *Nox2*^−/−^*Kras*^WT^ mice. Statistics by Student's *t* test. *p < 0.05, **p < 0.01, ***p < 0.001

To determine effects of in vivo treatment with *N*MH on the formation of ROS in accumulating myeloid leukemic cells, peripheral blood was recovered from control and *N*MH-treated M-*Kras*^G12D^ mice. Myeloid cells were analyzed for intracellular ROS content using the 2',7' -dichlorofluorescin diacetate (DCFDA) probe. It was observed that the intracellular ROS levels in CD11b^+^ myeloid cells increased significantly during disease progression (Fig. 4a). In accordance, myeloid cells isolated from the bone marrow and spleen of endpoint *Kras*^G12D^ mice showed significantly higher DCFDA staining compared with myeloid cells from *Kras*^WT^ bone marrow and spleen (Supplementary figure 3A, D). Furthermore, systemic treatment with *N*MH significantly reduced the accumulation of ROS in KRAS-expressing myeloid cells at 3 and 5 weeks after pIpC injections (Fig. 4a). Myeloid cells from *Nox2*^−/−^ M-*Kras*^G12D^ mice expressed significantly lower intracellular ROS levels compared with M-*Kras*^G12D^ mice with intact NOX2 at 5 weeks after pIpC injection (Fig. 4d), and the ROS levels were unaffected by systemic *N*MH treatment in these triple transgenic mice (Fig. 4d). In addition, myeloid cells from endpoint *Nox2*^−/−^ M-*Kras*^G12D^ mice expressed significantly lower levels of intracellular ROS compared with *Nox2*^+/+^ M-*Kras*^G12D^ mice at a corresponding stage of disease (Supplementary figure 3A, D).

### Treatment with *N*MH does not impact on NK cell or T cell counts in M-*Kras*^G12D^ mice

Pharmacological inhibition of NOX2 was previously shown to reduce the release of extracellular ROS and thereby protect adjacent anti-neoplastic lymphocytes such as NK cells and T cells from ROS-induced inactivation [33, 34]. To investigate if the reduced ROS levels in myeloid cells following *N*MH treatment translated into improved aspects of immunity, we monitored T cells and NK cells in peripheral blood during the course of disease. These populations were also analysed in the bone marrow and spleen of mice at the endpoint of the experiment. It was observed that the percentage of NK cells and CD8^+^ T cells in blood of M-*Kras*^G12D^ mice decreased during disease progression. In vivo treatment with *N*MH did not significantly impact on the reduction of NK cells or T cells during the progression of leukemia (Supplementary figure 4B and D). At the end of the experiment, there were significantly fewer CD8^+^ T cells and NK cells in the spleen of M-*Kras*^G12D^ mice compared with WT mice (Supplementary figure 4C, E).

### In vivo treatment with *N*MH maintains genomic integrity and reduces oxidative stress in M-*Kras*^G12D^ mice

We next assessed whether the reduction in intracellular ROS levels following treatment of mice with *N*MH translated into a reduced mutation frequency in M-*Kras*^G12D^ mice. Guanine bases in DNA are sensitive to oxidation and may, when exposed to ROS, form 8-hydroxy-2’-deoxyguanosine (8-OHdG). As 8-OHdG tends to bind to thymidine rather than cytosine during replication, the level of 8-OHdG is regarded as a biomarker of oxidant-induced mutagenesis [35]. We therefore used serially recovered peripheral blood myeloid cells from control or *N*MH-treated M-*Kras*^G12D^ mice for analysis of 8-OHdG. The level of DNA oxidation was significantly lower in *N*MH-treated animals 3 weeks after pIpC injections (Fig. 4b).

We next investigated if the reduced DNA oxidation in myeloid cells following *N*MH treatment translated into protection against DNA double-stranded breaks (DSB). As the formation of DSB entails phosphorylation of the histone H2AX, the resulting phosphorylated protein gamma-H2AX reportedly reflects DSB [36]. Peripheral blood samples from *N*MH-treated and control M-*Kras*^G12D^ mice were analyzed for gamma-H2AX expression. Similarly to the 8-OHdG results, there was a decrease in the amount of DSBs in the blood of *N*MH-treated mice that reached significance at 5 weeks after pIpC injections (Fig. 4c).

Myeloid cells isolated from the bone marrow and spleen of endpoint M-*Kras*^G12D^ mice showed excess 8-OHdG and gamma-H2AX expression staining compared with bone marrow and spleen cells of *Kras*^WT^ littermates and compared with bone marrow and spleen cells from endpoint *Nox2*^−/−^ M-*Kras*^G12D^ mice. 8-OHdG and gamma-H2AX expression levels in bone marrow and spleen cells did not differ between *N*MH-treated and control M-*Kras*^G12D^ mice at the endpoint of leukemia progression (Supplementary figure 3).

## Discussion

*KRAS* mutations in myeloid cells are associated with myeloproliferative disease in humans and mice. As mutated RAS has proven difficult to target directly [10], strategies to inhibit cellular functions that are induced by oncogenic RAS is a conceivable alternative in treating RAS-related leukemogenesis. In this study, we assessed the anti-leukemic properties of NOX2 inhibition in mice carrying *Kras*^G12D^ in hematopoietic cells (M-*Kras*^G12D^). A first finding was that myeloid cells that accumulated in M-*Kras*^G12D^ expressed functional NOX2 and thus produced significant amounts of ROS. In vitro studies showed that *N*MH, a histamine analogue with affinity at H_2_R, significantly reduced ROS formation in the expanding leukemic cells acting via H_2_R.

During the progression of leukemia, the ROS levels of the myeloid cells gradually increased. Treatment of M-*Kras*^G12D^ mice with *N*MH reduced the formation of ROS in vivo and significantly delayed the expansion of leukemia along with prolonging survival. At the endpoint of leukemia progression, there was no difference between ROS levels in myeloid cells of bone marrow or spleen of *N*MH-treated and control M-*Kras*^G12D^ mice, which likely is explained by the limited duration of treatment (5 weeks) and by the prolonged survival of *N*MH-treated mice. In addition, myeloproliferation was markedly reduced in *Nox2*^−/−^ M-*Kras*^G12D^ mice and the anti-leukemic properties of *N*MH were absent in these triple transgenic mice. In parallel, in vivo treatment with *N*MH as well as the genetic depletion of *Nox2* significantly reduced DNA oxidation and DSB in mice carrying M-*Kras*^G12D^.

These results imply that formation of NOX2-derived ROS contributes to leukemia and mutagenesis in M-*Kras*^G12D^ mice and that the targeting of NOX2 represents a conceivable anti-leukemic strategy. We cannot exclude, however, that non-NOX2-derived sources of ROS contributed to the ROS accumulation and oxidative stress observed in the proliferating *Kras*^G12D^ myeloid cells. The conclusion that *N*MH exerted anti-leukemic efficacy by targeting NOX2 was based on its inhibitory action on NOX2-dependent ROS formation and on its lack of anti-leukemic efficacy in *Nox2*^−/−^ mice, but our findings do not rule out the contribution by additional or supplementary mechanisms.

Previous studies in experimental cancer models suggest a link between RAS activation and ROS derived from various NOX species or from mitochondrial respiration [21–23]. For example, RAS expression in fibroblasts was shown to stimulate the Rac-dependent assembly of NOX1 leading to superoxide anion production and enhanced cell cycling [15]. Furthermore AKT, a kinase downstream of RAS and PI3K signaling, was shown to induce NOX4 expression and thus enhance melanoma cell proliferation [37]. However, activation of Rac or AKT may also stimulate NOX2-derived superoxide production [38, 39] and in the myeloid leukemia model employed in the present study the ROS levels were reduced by > 50% in *Nox2*^*−/−*^ vs. *Nox2*^*+/+*^*Kras*^G12D^ myeloid cells. Hence, NOX2 appears to be a dominant source of ROS in myeloid cells in the *Kras*^G12D^ model of myeloproliferative disease.

Unexpectedly, only pharmacological, but not genetic, inhibition of NOX2 prolonged the survival of M-*Kras*^*G12D*^ mice. This finding may imply that the complete absence of NOX2 characteristic of *Nox2*^−/−^ mice entails a survival disadvantage in KRAS-related leukemia. NOX2 is a pivotal component in the defense against several microbial agents [40] and indeed, the triple transgenic mice showed increased incidence of visible infections. Although other mechanisms may be operable, we hypothesize that the lack of impact of genetic depletion of NOX2 on the survival of M-*Kras*^*G12D*^ mice was related to infections that escaped detection.

Our findings add to a growing body of evidence, suggesting that the targeting of the formation of NOX2-derived ROS entails reduction of malignant tumor growth in vivo [41–44]. Although scavengers of ROS have shown discordant results by either promoting or inhibiting tumor cell growth in vivo [45–48], the specific targeting of NOX2 has been reported to reduce murine tumor growth, albeit with variable efficiency [41, 45–49]. The mechanisms by which NOX2 inhibition impacts on tumor growth are likely multi-factorial. For example, in a melanoma model of lung metastasis, NOX2^+^ myeloid cells were found to accumulate in lungs to reduce the anti-metastatic action of lung-infiltrating NK cells by generating immunosuppressive extracellular ROS. In this setting, NOX2 inhibition rescued NK cells from ROS-induced inactivation and decreased metastasis formation by favoring immune-mediated clearance of melanoma cells [42]. Inhibition of NOX2-derived ROS has also been implicated in the differentiation and maturation of myeloid cells [41], and experiments using immunodeficient mice imply that inhibition of NOX2 reduces expansion of xenografted human cancer cells also in the absence of functional lymphocyte-mediated immunity [50, 51]. In addition, ROS, including NOX2-derived ROS, have been implicated in enhancing cell cycle proliferation and in increasing mutagenesis [52–54].

Although details regarding the anti-leukemic action of NOX2 inhibition in *Kras*-mutated mice remain to be defined, the phenotypic analysis of NK cells and T cells did not support improved immune surveillance during NOX2 inhibition in this model. Instead, our results point toward protection against ROS-induced oxidation of DNA in myeloid cells as a principal mechanism explaining the anti-leukemic activity of genetic and pharmacological targeting of NOX2. This assumption is in line with previous studies showing that treatment with *N*-acetyl cysteine, which exerts antioxidant activity by augmenting cellular thiols, reduces DSBs and facilitates non-homologous end joining repair in bone marrow cells in a murine NRAS/BCL2-related model of leukemia [22]. Also, other genetic alterations that entail aberrant activation of signal transduction pathways, including *BCR-ABL* in chronic myeloid leukemia and *FLT3/ITD* in AML, are associated with elevated ROS formation in hematopoietic cells [19, 55], and enhanced levels of intracellular ROS have been proposed to enhance double-stranded DNA breaks and genomic instability to propagate leukemia also in these myeloid malignancies [20, 21].

In conclusion, the results of this study suggest (i) that NOX2-derived ROS may contribute to leukemic expansion in mice carrying hematopoietic cells with mutated *Kras* and (ii) that strategies to target NOX2 merit further evaluation in *RAS*-mutated leukemia.

## Materials and methods

### Genetically modified mice and experimental design

B6.129S4-Kras^tm4Tyj^/J, B6.Cg-Tg(Mx1-cre)1Cgn/J, and B6.129S6-*Cybb*^*tm1Din*^ mice were obtained from Jackson Laboratories (USA) and maintained under specific pathogen-free conditions. B6.129S4-Kras^tm4Tyj^/J mice carry a Lox-Stop-Lox (LSL) termination sequence followed by the *Kras*^G12D^ point mutation. When bred to the B6.Cg-Tg(Mx1-cre)1Cgn/J strain, which expresses Cre recombinase under the control of the Mx1 promoter, i.p. injection of pIpC (Sigma-Aldrich, St Louis, USA) induces endogenous interferon production that activates the Cre recombinase in hematopoietic cells with ensuing deletion of the transcriptional termination sequence, allowing for expression of oncogenic *Kras* in hematopoietic cells (M-*Kras*^G12D^ mice) [30].

To study the role of NOX2 in this model, B6.129S6-*Cybb*^*tm1Din*^ (*Nox2*^−/−^*)* mice that lack the myeloid gp91^phox^ subunit NOX2, and thus a functional NOX2, were bred to B6.129S4-Kras^tm4Tyj^/J mice to generate *Nox2*^−/−^*Kras*^G12D^ mice and to Mx1-*Cre* mice to generate *Nox2*^−/−^ Mx1-*Cre* mice. These mice were backcrossed at least three times to achieve offspring with close genetic identity. Finally, *Nox2*^−/−^*Kras*^G12D^ mice and *Nox2*^−/−^ Mx1-*Cre* mice were mated to generate *Nox2*^−/−^ LSL-*Kras*^G12D^ Mx1-*Cre* (*Nox2*^−/−^ M-*Kras*^G12D^) mice. Lack of functional NOX2 in these mice was confirmed by genotyping for *Nox2* using PCR and by measuring the lack of NOX2-dependent ROS production with chemiluminescence (Supplementary figure 1A, B).

Genotyping, as detailed below, was employed to identify double and triple transgenic pups. Three to four weeks old double transgenic M-*Kras*^G12D^ or triple transgenic *Nox2*^−/−^ M-*Kras*^G12D^ mice received three doses of pIpC (250 μg) i.p. every second day starting 1 week after the first pIpC injection and continuing for 5 weeks, M-*Kras*^G12D^ or *Nox2*^−/−^ M-*Kras*^G12D^ mice were treated with *N*MH (Sigma) (250 μg/mouse) i.p. every second day using vehicle-treated mice (NaCl) as controls. Activation of a recombined *Kras*^G12D^ allele was confirmed by PCR analysis of peripheral blood 2 weeks after pIpC injections. Mice were weighed and blood was collected every second week to follow the course of disease. Blood counts were determined on a Sysmex KX-21 Hematology Analyzer (Sysmex, Kobe, Japan). When moribund, mice were anesthetized and killed by cervical dislocation followed by recovery of spleen, bone marrow, and thymus. All experiments were approved by the Research Animal Ethics Committee at the University of Gothenburg, Sweden (application no. 86/14).

### Genotyping

Genomic DNA was extracted from mouse ear biopsies of 3-week-old pups for PCR analysis using the mouse direct PCR kit (Biotool, Houston, USA). The following primers were used for detection of the *Nox2*^−/−^ allele, *Kras2*^LSL^ allele, *Kras2*^G12D^ allele and the Mx1-*Cre* allele:

F-*Nox2*^−/−^ and *Nox2*^WT^: AAGAGAAACTCCTCTGCTGTGAA

R-*Nox2*^WT^: CGCACTGGAACCCCTGAGAAAGG

R-*Nox2*^−/−^: GTTCTAATTCCATCAGAAGCTTATCG

F-*Kras*^G12D^: CCTTTACAAGCGCACGCAGACTGTAGA

R-*Kras*^G12D^: AGCTAGCCACCATGGCTTGAGTAAGTCTGCA

F-Mx1-*Cre*: GTTTCAATTCTCCTCTGGAAGG

R-Mx1-*Cre*: CTAGAGCCTGTTTTGCACGTTC

F-*Kras*^LSL^: TCCGAATTCAGTGACTACAGATGTACAGAG

R-*Kras*^LSL^: GGGTAGGTGTTGGGATAGCTG

### Cell preparation

Single-cell suspensions of splenocytes and thymic cells were prepared by mashing tissues through a 70-μm cell strainer. For the isolation of bone marrow cells, the femur, tibia, and hip bones were mechanically cleaned from surrounding tissue and rinsed briefly in 70% ethanol followed by phosphate-buffered saline (PBS). The clean bones were crushed with mortar and pestle in PBS. The cell mixture was filtered through a 40-μm filter and washed three times in PBS. Erythrocytes in the cell lysates were depleted using the RBC Lysing buffer (Sigma-Aldrich, Steinheim, Germany). Gr1^+^ cells, at a minimum of 95% purity, were isolated from single-cell spleen suspensions using biotinylated Gr1 antibodies followed by streptavidin-conjugated MACS beads (Miltenyi Biotec, Lund, Sweden) according to the manufacturer’s instructions.

### Flow cytometry

The following fluorochrome-labeled anti-mouse mAbs were, unless otherwise stated, purchased from BD Biosciences: anti-CD11c (HL3), anti-IaIe (2G9), anti-CD3(145-2311), anti-CD4 (RM4-5), anti-CD8 (53-6.7), anti-NK1.1 (PK136), anti-CD335 (29A1.4-eBiosciences), anti-CD19 (1D3), anti-CD11b (M1/70), anti-Gr1 (RB6-8C5), anti-F4/80 (BM8-Biolegend) and anti-Ly6C (AL-21). In addition, LIVE/DEAD® Fixable Yellow Dead Cell Stain Kit (Invitrogen) and diamidino-2-phenylindole (DAPI) (Invitrogen) were used for flow cytometric assessment. A minimum of 100,000 gated live cells were collected on a five-laser BD LSRFortessa (355, 405, 488, 532, and 640 nm; BD Biosciences). Data were analyzed using the FACSDiva Version 8.0.1 software (BD Biosciences).

### Detection of ROS by chemiluminescence

Superoxide anion production was determined by the use of isoluminol-ECL, as previously described [56]. Gr1^+^ cells were suspended to 20 × 10^4^ cells/ml in Krebs-Ringer glucose buffer supplemented with isoluminol (10 mg/ml; Sigma-Aldrich) and horseradish peroxidase (4 U/ml; Boehringer Mannheim, Mannheim, Germany) and added to a 96-well plate. The formyl peptide receptor agonist WKYMVm (10^−7^ M Tocris Bioscience, Bristol, UK) was added to stimulate NOX2-derived ROS production, and light emission reflecting superoxide production was recorded continuously using a FLUOstar Omega plate reader (BMG, Ortenberg, Germany). These experiments were performed in the presence or absence of *N*MH, added 5 min before the addition of WKYMVm. In some experiments, the histamine H_2_R antagonist ranitidine (Glaxo, Gothenburg, Sweden) was added prior to *N*MH stimulation to ensure the receptor specificity of *N*MH.

### Detection of ROS by DCFDA

Peripheral blood or bone marrow cells were first stained for expression of CD11b (M1/70) using a fluorochrome-conjugated antibody. The cells were then incubated 30 min at 37 °C with 4.7 μM DCFDA in RPMI 1640 medium (Merck Millipore, Berlin, Germany). Cells were washed and analyzed by flow cytometry in the presence of DAPI for exclusion of dead cells.

### Detection of DNA strand breaks and oxidated DNA

Peripheral blood or bone marrow cells were fixed and permeabilized for 20 min with the fixation/permeabilization concentrate (eBioscience^TM^, San Diego, USA). Following washing in permeabilization buffer twice, cells were incubated at room temperature for 30 min with anti-H2AX (pS139; BD Biosciences) for detection of DSA or an anti-8 hydroxyguanosine antibody (Abcam) for detection of oxidative stress on DNA [35, 36]. Data were collected on a five-laser BD LSRFortessa (355, 405, 488, 532, and 640 nm; BD Biosciences).

### Statistical analysis

For the animal experiments, the sample size was dependent on the availability of mice of a similar age and with a specified genetic profile; hence double transgenic (LSL-*Kras*^G12D^ *×* Mx1-*Cre*) and in particular triple transgenic (*Nox2*^−/−^ LSL-*Kras*^G12D^ × Mx1-*Cre*) mice. All in vivo experiments were performed at least three times.

Animals were randomly assigned to treatment or control groups and codes were ascribed to each animal. Analyses were performed in a single blinded fashion, as the investigator assessing the treatment outcome was not aware of which mouse that received which treatment. According to ethical regulations, mice with visible infections were killed and thus excluded from analysis.

Graphpad Prism (version 7, San Diego, CA) was used for statistical analysis. For group comparisons, two-tailed paired or unpaired *t* tests were used. For multiple comparisons one-way analysis of variance was used followed by the Bonferroni multiple comparison test. All statistical tests were two-sided, and − values below 0.05 were considered statistically significant.

## Electronic supplementary material

Supplementary figure 1

Supplementary figure 2

Supplementary figure 3

Supplementary figure 4
