# Supplementary material for: NOX2 inhibition reduces oxidative stress and prolongs survival in murine KRAS-induced myeloproliferative disease
Source: Oncogene. 2018 Oct 15;38(9):1534–43. doi: 10.1038/s41388-018-0528-1 (PMC6372471; doi:10.1038/s41388-018-0528-1)
Supplement: Supplementary file 1 — Supplementary figure 1 [file 41388_2018_528_MOESM1_ESM.pdf]

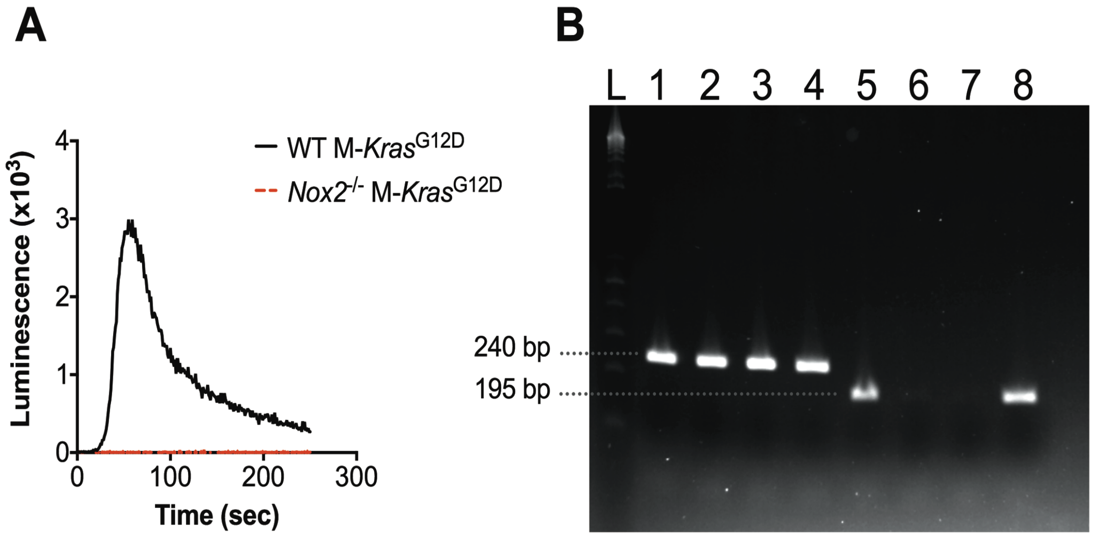

Supplementary Fig. 1. Triple transgenic *Nox2*$^{-/-}$ M-*Kras*^{G12D} mice are devoid of ROS production. (A) ROS production measured by chemiluminescence in splenic Gr1^{+} cells isolated from WT or *Nox2*$^{-/-}$ M-*Kras*^{G12D} mice following WKYMVm (10^{-7} M) stimulation. (B) Gel picture showing presence of *Nox2* in WT mice (lanes 1-4), presence of mutant *Nox2* in *Nox2*$^{-/-}$ M-*Kras*^{G12D} mice (lanes 5 and 8) and absence of WT *Nox2* in *Nox2*$^{-/-}$ M-*Kras*^{G12D} mice (lanes 6 and 7), as measured by PCR on peripheral blood.
