# Supplementary material for: NOX2 inhibition reduces oxidative stress and prolongs survival in murine KRAS-induced myeloproliferative disease
Source: Oncogene. 2018 Oct 15;38(9):1534–43. doi: 10.1038/s41388-018-0528-1 (PMC6372471; doi:10.1038/s41388-018-0528-1)
Supplement: Supplementary file 2 — Supplementary figure 2 [file 41388_2018_528_MOESM2_ESM.pdf]

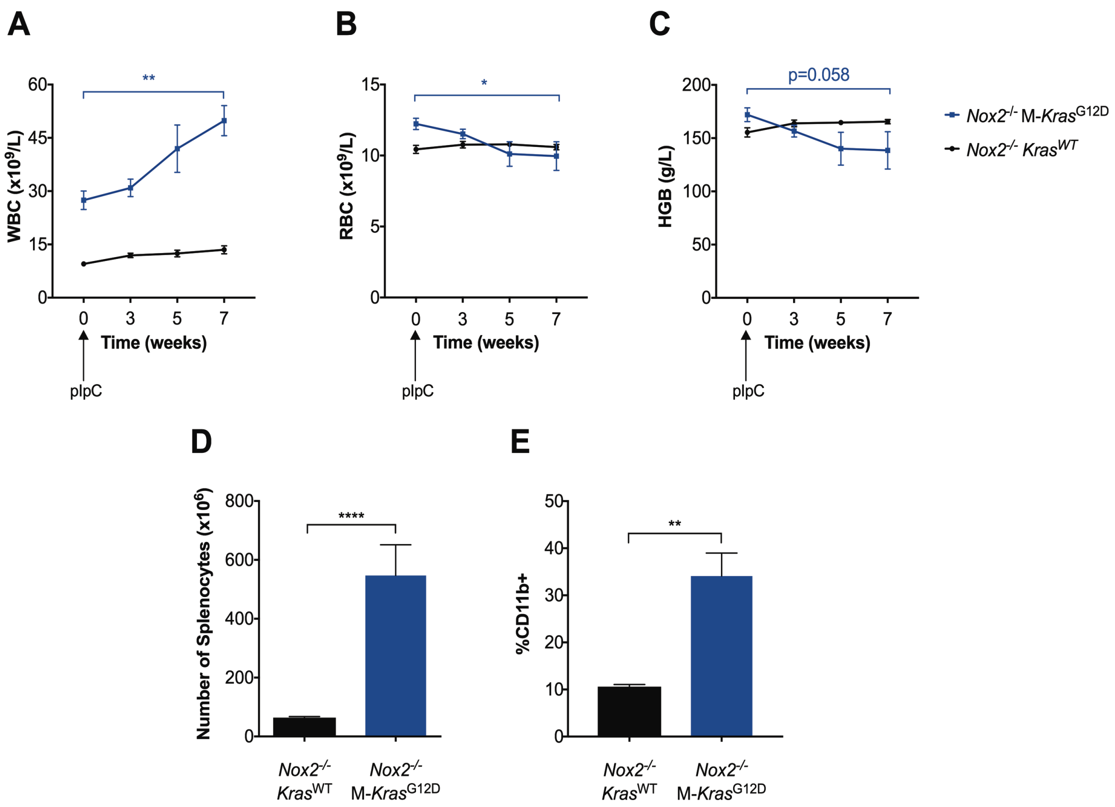

Supplementary Fig. 2. Characterization of *LSL-Kras*^{G12D} × Mx1-*Cre*-induced leukemia in *Nox2*$^{-/-}$ mice. (A-C) *Nox2*$^{-/-}$LSL-*Kras*^{G12D} × *Nox2*$^{-/-}$Mx1-*Cre* (*Nox2*$^{-/-}$ M-*Kras*^{G12D}; blue lines) mice and wild-type (*Nox2*$^{-/-}$*Kras*^{WT}; black lines) mice were injected with pIpC at the time point w_0. Peripheral blood was collected at 3, 5 and 7 weeks (w3-7) after pIpC injection. The peripheral blood of *Nox2*$^{-/-}$M-*Kras*^{G12D} mice showed a progressive increase in white blood cell (WBC) counts (A), along with a reduction of red blood cell (RBC) counts (B) and hemoglobin levels (C; n=8 for *Nox2*$^{-/-}$*Kras*^{WT} at each time point, n=8 for *Nox2*$^{-/-}$M-*Kras*^{G12D} at each time point except n=2 at w7). (D) Number of splenocytes isolated from endpoint *Nox2*$^{-/-}$M-*Kras*^{G12D} mice (blue, n=6). The splenocyte counts from *Nox2*$^{-/-}$*Kras*^{WT} mice (black, n=6) are shown for comparison (*t*-test). (E) Frequency of CD11b^{+}-infiltrating cells among live cells in the spleens of endpoint *Nox2*$^{-/-}$M-*Kras*^{G12D} mice (blue, n=5) and *Nox2*$^{-/-}$*Kras*^{WT} mice (black, n=5) (*t*-test). *$p<0.05$, **$p< 0.01$, ***$p< 0.001$.
