# Supplementary material for: NOX2 inhibition reduces oxidative stress and prolongs survival in murine KRAS-induced myeloproliferative disease
Source: Oncogene. 2018 Oct 15;38(9):1534–43. doi: 10.1038/s41388-018-0528-1 (PMC6372471; doi:10.1038/s41388-018-0528-1)
Supplement: Supplementary file 3 — Supplementary figure 3 [file 41388_2018_528_MOESM3_ESM.pdf]

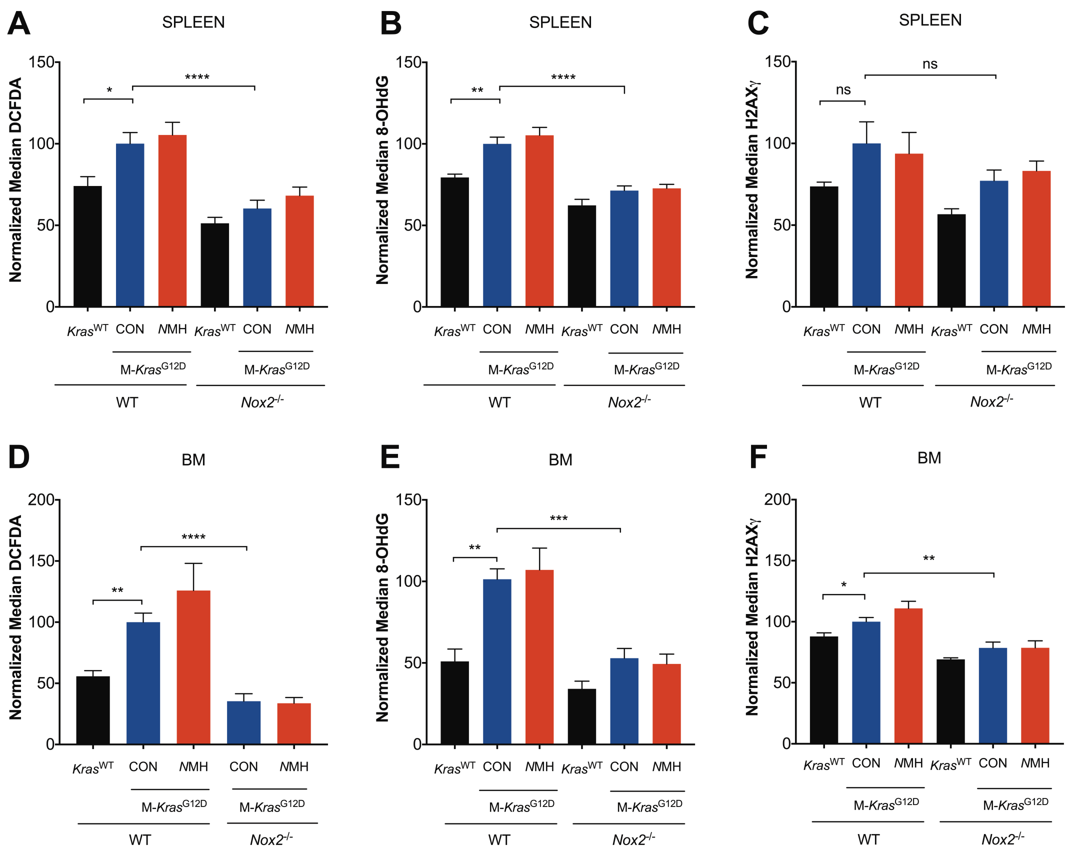

Supplementary Fig. 3. Oxidative stress, DNA oxidation and DSB levels in the BMs and spleens at the endpoint. (A-F) Expression of mutated *Kras* in hematopoietic cells was induced in double (M-*Kras*^{G12D}) and triple (*Nox2*$^{-/-}$M-*Kras*^{G12D}) transgenic mice by pIpC injections. Mice were treated with *N*MH (250 µg/mouse; red) or NaCl (CON; blue) i.p. thrice weekly for 5 weeks. When moribund, spleen (A-C) and bone marrow (D-F) cells were collected from them M-*Kras*^{G12D} and *Nox2*$^{-/-}$M-*Kras*^{G12D} mice and analyzed for (A and D) DCFDA expression in CD11b^{+} cells (B and E) 8-OHdG expression and (C and F) gamma-H2AX expression in myeloid cells (for spleens: n=8 for *Kras*^{WT}, n=12 for M-*Kras*^{G12D}, n=8 for *Nox2*$^{-/-}$ *Kras*^{WT}, n=7-8 for *Nox2*$^{-/-}$ M-*Kras*^{G12D}; for BMs: n=4-7 for *Kras*^{WT}, n=8-12 for M-*Kras*^{G12D}, n=3 for *Nox2*$^{-/-}$*Kras*^{WT}, n=5-6 for *Nox2*$^{-/-}$ M-*Kras*^{G12D}). Student´s *t*-test. *p<0.05, **p<0.01, ***p< 0.001.
