# Supplementary material for: NOX2 inhibition reduces oxidative stress and prolongs survival in murine KRAS-induced myeloproliferative disease
Source: Oncogene. 2018 Oct 15;38(9):1534–43. doi: 10.1038/s41388-018-0528-1 (PMC6372471; doi:10.1038/s41388-018-0528-1)
Supplement: Supplementary file 4 — Supplementary figure 4 [file 41388_2018_528_MOESM4_ESM.pdf]

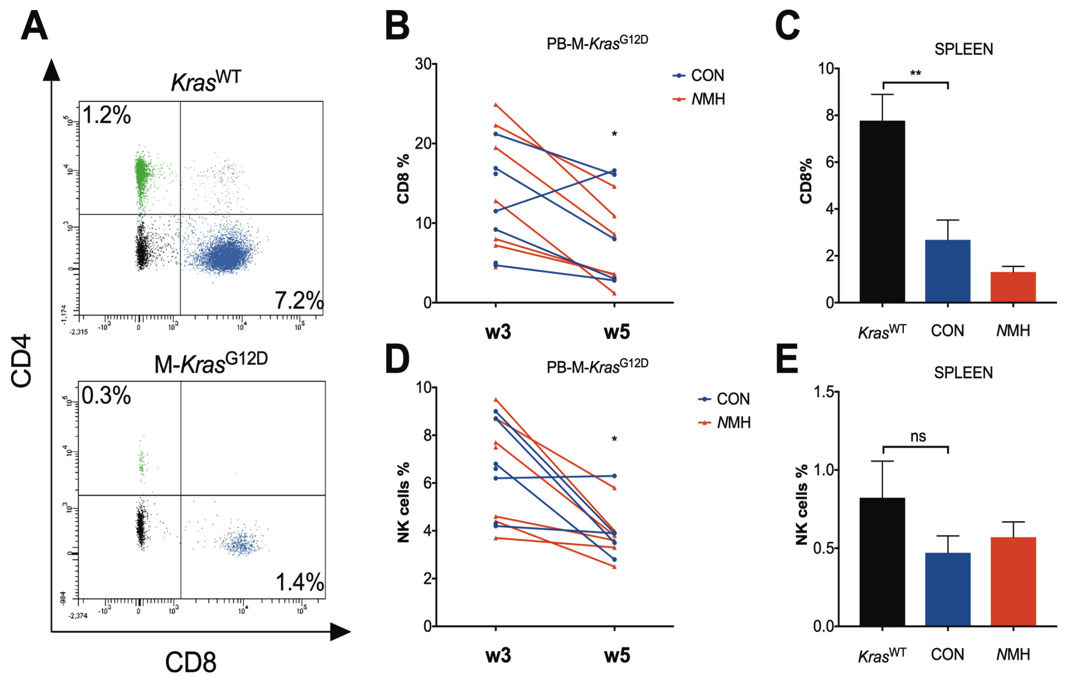

Supplementary Fig. 4. T cell populations in PB and spleens of *LSL-Kras*^{G12D} × Mx1-*Cre*-induced leukemic mice. LSL-*Kras*^{G12D} × Mx1-*Cre* (M-*Kras*^{G12D}) mice were injected with pIpC at the time point w_0. Mice were treated with *N*MH (250 µg/mouse; red) or NaCl (CON; blue) i.p. thrice weekly for 5 weeks. (A) A representative FACS plot showing CD4$^+$ and CD8$^+$ T cells in the spleen of a representative endpoint M-*Kras*^{G12D} mouse and a *Kras*^{WT} mouse. (B, D) Peripheral blood collected at 3 and 5 weeks (w3-5) after pIpC injection showed a significant decrease in frequency of (B) CD3$^+$CD8$^+$ T cells and (D) NKp46$^+$ NK cells among CD45$^+$ live cells in M-*Kras*^{G12D} mice. (C, E) When moribund, splenocytes were collected from the sacrificed M-*Kras*^{G12D} mice and were analyzed for (C) T cell content and (E) NK cell content. (n=9 for *Kras*^{WT}, n=10 for control and *N*MH treated M-*Kras*^{G12D}). Student´s t-test. *p<0.05, **p<0.01, ***p< 0.001.
